# The Effect of Feeding Management and Culling of Cows on the Lactation Curves and Milk Production of Primiparous Dairy Cows

**DOI:** 10.3390/ani11071959

**Published:** 2021-06-30

**Authors:** Jolanta Różańska-Zawieja, Stanisław Winnicki, Joanna Zyprych-Walczak, Alicja Szabelska-Beręsewicz, Idzi Siatkowski, Włodzimierz Nowak, Barbara Stefańska, Ryszard Kujawiak, Zbigniew Sobek

**Affiliations:** 1 Department of Genetics and Animal Breeding, Poznan University of Life Sciences, ul. Wołyńska 33, 60-637 Poznań, Poland; jolanta.rozanska-zawieja@up.poznan.pl; 2 Institute of Technology and Life Sciences, Poznań Branch, ul. Biskupińska 67, 60-463 Poznań, Poland; st-winnicki@wp.pl; 3 Department of Mathematical and Statistical Methods, Poznan University of Life Sciences, ul. Wojska Polskiego 28, 60-637 Poznań, Poland; joanna.zyprych-walczak@up.poznan.pl (J.Z.-W.); alicja.szabelska-beresewicz@up.poznan.pl (A.S.-B.); idzi.siatkowski@up.poznan.pl (I.S.); 4 Department of Animal Nutrition, Poznań University of Life Sciences, ul. Wołyńska 33, 60-637 Poznań, Poland; wlodzimierz.nowak@up.poznan.pl (W.N.); barbara.stefanska@up.poznan.pl (B.S.); 5 Department of Grassland and Natural Landscape Sciences, Poznań University of Life Sciences, ul. Dojazd 11, 60-632 Poznań, Poland; 6 Sano-Modern Animal Feeding, Lubiń, ul. Lipowa 10, 64-541 Sękowo, Poland; rku@sano.pl

**Keywords:** primiparous cows, lactation curves, feeding system, herd management

## Abstract

**Simple Summary:**

Curves for milk yield and milk constituents were plotted according to Wood’s model. The curves were compared for four herds with a high milk yield (over 10,000 kg). The farms used different feeding systems. Differences were noted in the number of days in milk at which the herds reached the peak of lactation. The herd that reached peak production latest had the highest peak milk yield as well as the highest average milk yield for the entire lactation. The effect of early culling of primiparous cows after 30, 60 and 90 days in milk was analysed in herd T1. Such early culling of primiparous cows prevents their negative impact on the average yield of the entire herd. This increases the chance of improving yield in the herd by quickly introducing new, genetically more valuable cows.

**Abstract:**

The study attempted to estimate the lactation curves of primiparous dairy cows in relation to their feeding management. Therefore, the first aim of the study was to determine and compare the lactation curves of primiparous dairy cows using Wood’s model and to estimate the association between the lactation curves and feeding management. The second objective was to investigate the effect of the culling rate on improvement in the milk yield of primiparous dairy herds. The study was conducted on four commercial dairy farms of Polish Holstein–Friesian cows using different feeding systems (TMR—total mixed ration and PMR—partial mixed ration) and management (T1—one TMR throughout lactation; P1—one PMR throughout lactation; T2 and T3—three feed periods such as FRESH, TMR I and TMR II according to days in milk). The data used for the study were obtained from monthly milk performance evaluations of 1662 primiparous cows conducted by the Polish Federation of Cattle Breeding and Dairy Farmers throughout the year 2015. Wood’s lactation model was used to plot curves for milk yield, fat and protein content, lactose content, and milk urea contents. The highest milk yield for the whole lactation and in the peak lactation phase was recorded for cows in herd T1. This herd reached peak lactation on day 105 of milking, with an average milk yield of 42.1 kg, which was about 5 kg more milk than in the other herds. The study showed that the culling of primiparous cows in herd T1 after 30, 60 and 90 days of lactation prevented a significant reduction in milk yield in a 305-day lactation. It also increased average milk production by 1586.9 kg per primiparous dairy cow.

## 1. Introduction

Lactation curves are graphical representations of the course of lactation, taking into account all factors that affect milk production. The lactation curve illustrates the occurrence of changes in milk during lactation, involving a decrease in milk yield accompanied by an increase in fat and protein content [1]. The standard lactation curves of dairy cows show a maximum daily milk yield between 4 to 8 weeks after calving, followed by a daily decrease in yield until the dry period [1]. It is important for dairy cow farmers to know what level of milk yield the cow will reach the peak of lactation and how long after calving this peak will be observed.

There are currently many models and types of lactation curves in the literature. Common lactation models include the Ali–Schaeffer model [2], the Wilmink model [3], and the Wood model [4], which is the most popular. Analysis of lactation curves suggests that a flattened curve resulting from uniform lactation, during which milk yield remains at the same level for an extended period, is the most desirable. This is closely linked to lactation persistence, i.e., the rate at which milk production drops after the peak of lactation; the slower drop in production, the higher the persistence, which is also economically beneficial. Cows with higher lactation persistence ensure greater economic profit. It is assumed that milk production should decrease by 8–10% per month, with a 5% more rapid decrease in primiparous cows than in multiparous cows [5,6]. Moreover, higher milk yield could be associated with fertility, a 56-day increase in the calving-to-first-service interval [7]. Numerous studies also demonstrate that the lactation curve of primiparous cows has a lower peak and is more persistent than that of multiparous cows [8]. After the peak of lactation, milk production gradually decreases, but the dynamics of this process depend on many factors, nutrition, breed, age, diseases, frequency of milking, and length of dry period [9,10,11,12]. The productive performance of a herd is influenced by the level of culling of cows in successive months of lactation. Juszczak et al. (2003) reported that the cost of milk produced by cows used only in the first three lactations is equal to its market value, and only when used for a longer time do they begin to generate profit [13]. Therefore, early culling of cows will prevent them from reaching their production peak [14]. However, there are still limited scientific data on how lactation curves of primiparous dairy cows are influenced by feeding management and culling rates. Therefore, the first aim of this study was to determine and compare the lactation curves of primiparous dairy cows depending on the feeding management in field conditions using Wood’s model. The second objective was to investigate the effect of the culling rate on the improvement in the milk yield of dairy herds.

## 2. Materials and Methods

### 2.1. Farms

The study was conducted on four commercial dairy farms of Polish Holstein–Friesian cows (Table 1) using different feeding systems (TMR—total mixed ration and PMR—partial mixed ration) and management (T1—one TMR throughout lactation; P1—one PMR throughout lactation; T2 and T3—three feed periods as FRESH, TMR I and TMR II according to days in milk). The farms were selected according to milk yield (about 11,000 kg/305 days lactation per cow), the size of the farm (more than 100 lactating dairy cows), housing of cows (only free-stall barns), and milking system (automated). The data used in the study included milk yield and chemical composition during 305 day of lactation from primiparous cows that calved in 2015. Milk production performance data from morning and afternoon milking during monthly milk performance evaluations were used. The evaluations were conducted by the Polish Federation of Cattle Breeding and Dairy Farmers.

Analysis of the effect of early culling on lactation yield was performed only for herd T1, because it was the largest and had the highest milk yield (kg). The herd consisted of 779 primiparous cows (Table 1), but only 628 cows, whose lactation lasted 1–305 days, were included in the analysis. The remaining 151 cows were not included in the calculations because they were milked for longer than 305 days.

### 2.2. Chemical Composition of Diets

The diets were balanced based on analysed content of nutrients using Dairy Max System software (Cargill, Kiszkowo, Poland) formulated according to National Research Council guidelines [15]. On all farms the cows were fed diets based on maize silage; alfalfa silage; beet pulp; grass silage or wilted, ensiled high-moisture maize grain; barley, wheat, triticale, and maize grain; rapeseed and soybean meals; and mineral and vitamin supplements. Using wet chemistry analysis, monthly forage, concentrate, TMR, and PMR samples were tested for dry matter (DM, method No. 6496), crude protein (CP, method No. 976.05), neutral detergent fibre (NDF, method No. 942.05), acid detergent fibre (ADF, method No. 973.18), non-fibre carbohydrates (NFC, method No. 64.785), starch (method No. 64.785), ether extract (EE, method No. 989.05), calcium (Ca, method No. 6869), phosphorus (P, method No. 6491), and potassium (K, method No. 6869) according to Procedures of the Association of Official Analytical Chemists [16]. Crude fibre was determined with an Ankom 220 fibre analyzer (ANKOM Technology, Macedon, NY, USA). TMR and PMR diets were administered at 9 a.m. and 2.30 p.m. throughout the experimental period (Table 1). 

### 2.3. Statistical Analysis

Estimation of lactation curves of primiparous cows based on test-day milk yield

The calculations were carried out using the R-package [17], with the following procedures: agricolae [18], easynls [19], ggplot2 [20], and openxlsx [21].

The lactation curves of primiparous cows for each trait (milk yield, fat and protein content, lactose content, and urea content) were determined according to Wood’s function [4], using the following model:

y = a × x^b^ × exp^−cx^

where:

y—a dependent variable determining the milk traits on the test day (day x)
x—number of days after calving
a—parameter determining the average milk traits on the test day
b—parameter determining the slope of the increasing part of the lactation function
c—parameter determining the slope of the decreasing part of the lactation function
exp—exponential function. For detailed interpretations of Woods function parameters [22,23].

For the lactation curves determined based on Wood’s function, milk yields were calculated using the definite integral (1) from calving to 305 days of milking; (2) from calving to lactation peak; (3) from calving to day of culling; (4) from calving to 305 days of milking for the curve estimated on the basis of shortened lactations. The coordinates x and y for the maximum value of y for this curve were calculated, as well as the areas under the curves, which correspond to the milk yields in the corresponding periods.

Since the data on milk yield curves were not subject to normal distribution (checked using the Shapiro–Wilk test), the Kruskal–Wallis test was used to compare the lactation curves instead of analysis of variance (ANOVA). A detailed comparison was made using Dunn’s non-parametric multiple comparison test with the Holma–Šidák correction [24,25,26].

To determine how the early culling of cows with a yield below the adopted criterion (24 kg milk) affects the total productivity of the herd, predicted yield was calculated for a situation without this culling, taking into account the numbers of cows in each period. If culled cows had been milked up to 305 days, without culling of those with the lowest production, the total 305 d yield for the primiparous cows in herd T1 would have been 9798.13 kg of milk. By eliminating cows whose yield was too low, the average yield in the herd was increased by 1586.87 kg of milk per cow.

To test for differences between feeding strategies (or farms) regarding peak milk yields or specific milk charts (up to 30, 60, 90, etc. days), we used the definite integrals for Wood’s function.

## 3. Results

**Figure 1:**
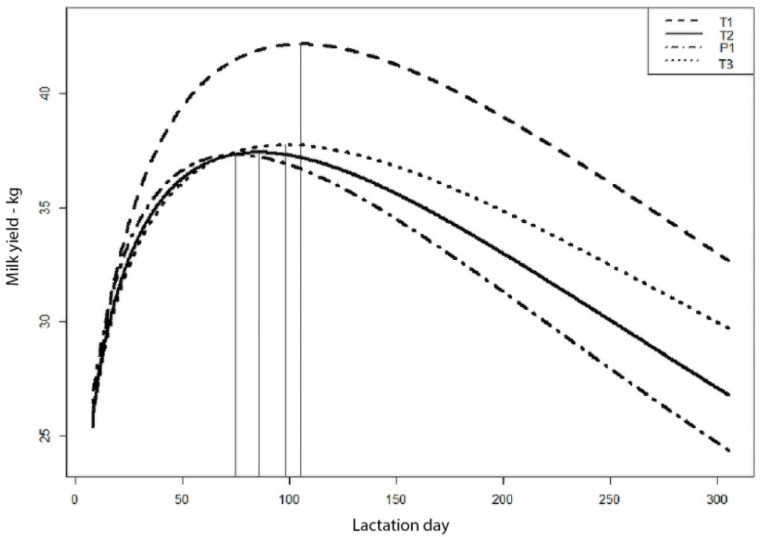
Lactation curves of primiparous cows with different feeding management.

The shape of lactation curves determined on the basis of average daily milk yields in all herds was consistent with the pattern generally accepted as normal. The flattest curve was obtained for the T3 herd (Figure 1). The lactation peak was noted on day 98 of lactation, and the average milk yield at that time was 37.8 kg. Similar milk yields (37.4 and 37.3 kg) at the peak of lactation were obtained for the cows in herds T2 and P1. The most rapid decrease in the lactation curve was observed in the P1 herd, in which the lactation peak was noted on day 75. The highest milk yield for the whole lactation and in the peak lactation phase was recorded for cows in herd T1. For the latest peak (105 days), this portion accounted for only 32.8% of the total lactation. Cows reached peak lactation in this herd on day 105 of milking, with an average milk yield of 42.2 kg, which was about 5 kg more milk than in the other herds.

Herds: T1—one TMR throughout lactation; P1—one PMR throughout lactation; T2 and T3—three feeding periods: FRESH, TMR I and TMR II, according to days in milk (kg).

The results show that the time to reach the peak of lactation was different in herds with different feeding management (Figure 1). Thus, the highest lactation yield was recorded in herd T1, in which the peak of lactation was reached the latest (on the 105th day in milk). Interestingly, a later peak of lactation was associated with a smaller decrease in productivity following the peak. The share of this fraction increases with earlier peaks in lactation. For peak on the 75th day, the share of this fraction increased to 36% in the P1 herd.

**Figure 2:**
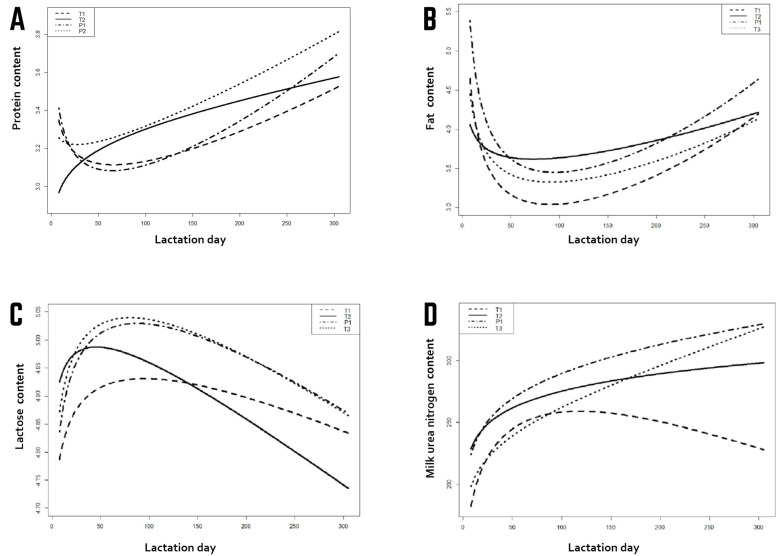
Presents the results for other milk traits in herds with different feeding management systems. Curves for protein (**A**), fat (**B**), lactose (**C**), milk urea (**D**) contents Herds: T1—one TMR (total mixed ration).throughout lactation; P1—one PMR (partial mixed ration) throughout lactation; T2 and T3—three feeding periods: FRESH, TMR I and TMR II, according to days in milk (kg).

A decrease in the protein content of milk up to 50–60 days in milk was observed in herds T1 and P1, after which the value of this parameter significantly improved. This curve was completely different for herd T2, in which there was no such decrease; the protein content in the milk was very low at the beginning of lactation and subsequently showed a marked increase. Additionally, analysis of the fat content of milk revealed a decline up to about day 100. A much slower decrease in the percentage content of fat was noted in the T1 system, only until about day 20–30 of milking. After this, it was relatively stable, with a slight upward trend. The curves for lactose content in milk for herds P1 and T3 were almost identical. The T1 herd had a much more stable lactose content, but at the lowest level. The T2 herd initially had the highest lactose content, but then it decreased the most sharply, reaching 4.75% at the end of lactation. In the P1 and T3 systems, the milk urea (MU) concentration increased significantly from the beginning of lactation and exceeded 350 mg/l at the end of lactation. In the T1 and T2 systems, the values were more stable, oscillating around 250 mg/L. In the case of herd T1, the curve for MU was similar to the curve for milk yield, with a maximum of about 100 days of lactation for both curves (Figure 2).

Analysis of the areas under the lactation curves in herd T1, determined by their intersection with the lines denoting 100, 200 and 305 days of lactation, revealed the highest percentage of the total area for the period from 100–200 days of lactation, whose share in the total lactation was 35.6–36%. This share of phase 2 in the total lactation yield indicates highly uniform milk production over the course of lactation.

The impact of culled primiparous cows on milk production was examined in herd T1. Predicted lactation yields were estimated on the basis of Wood’s function, increasing the number of days based on which this function was determined. The functions were used to estimate the expected 305 day yield. Knowing the actual milk yield of the T1 herd (11,385 kg) and the estimation of this yield based on initial data from the lactation, it can be concluded that the accuracy of the prediction increases with the number of days in milk (Table 2.)

**Figure 3:**
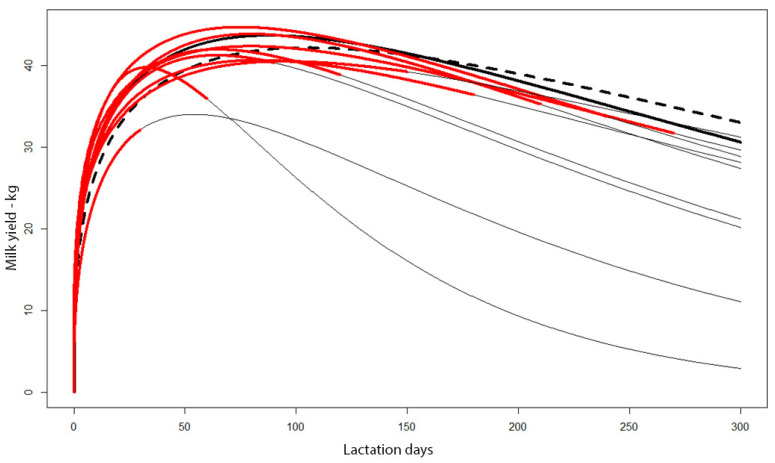
Milk yield curves for cows culled after day x of milking (red lines), extended based on predictions made using the Wood function (thin black lines). Total curve for cows milked for 305 days and those with shortened lactation (dashed line). Curve for cows milked at least 305 days (thick black line).

For herd T1 with very strict culling, curves were created for milk yields for actual days of milking of cows culled up to 30, 60, 90, etc. days. Figure 3 clearly indicates that for the shortened curves that reached the peak of lactation, the peak was very early and had a much lower value than for the rest of the herd, milked for 305 days.

If primiparous cows had not been culled due to low yields, this would clearly have reduced the lactation yield of the whole herd. To determine the losses that would have been caused by not culling these cows, a yield prediction using the Wood function was prepared (Figure 3). Curves for shortened lactations were compared with the total yield curve of the herd composed of previously culled cows and those that were milked until the end of lactation (thick black line). Additionally, a curve (Figure 3) was plotted (dashed line) for all cows except those culled before day 305 of lactation.

**Figure 4:**
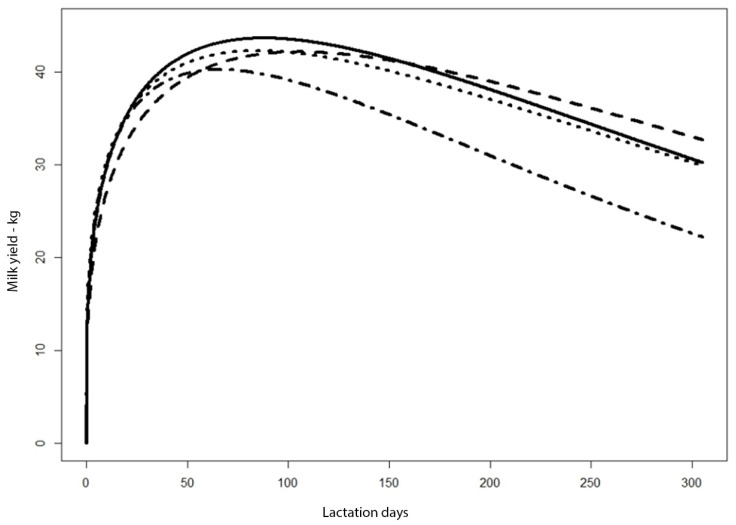
Predicted milk yield curves for culled primiparous cows. Legend: Dashed line—data for all cows milked in herd T1 up to day 305 (including culled cows). Continuous line—data only for cows milked up to day 305. Dotted line—data as for the dashed line, but excluding cows culled up to day 90. Dashed dotted line—data for average values from Figure 3.

Using the curves determined by the Wood function, the integrals from day 0 to day x of milking and milk yield predict for 305 days were calculated for each successive function. The values of these integrals correspond to the milk yield over the corresponding periods (Table 3). These calculations are confirmed by the graphic (Figure 4), suggesting that culling of cows after 30, 60 and 90 days of lactation prevented a significant reduction in the milk yield of the whole herd, to 7109.765, 5610.461 and 9717.556 kg of milk, respectively, for 305 days of milking.

The milk yield for the period of 0–305 days of milking was calculated for the curves from Figure 4. These values are similar and range from 11,348.95 to 11,614.94.

To determine how the early culling of cows with a yield below the adopted criterion (24 kg milk) affects the total production of the herd, predicted yield was calculated for a situation without this culling, taking into account the numbers of cows in each period. If culled cows had been milked up to 305 days, without culling of those with the lowest production, the total 305 d yield for the primiparous cows in herd T1 would have been 9798.13 kg of milk. By eliminating cows whose yield was too low, an additional 1586.87 kg of milk per cow was obtained in this herd.

The result of the Kruskal–Wallis test (*p* = 0.003) indicated that the curves differed statistically significantly. Dunn’s non–parametric test of simultaneous multiple comparisons with the Holma–Šidák correction was used for detailed comparisons. The results indicate that charts y_0_ and y_90–305_ do not differ significantly statistically (*p* = 0.71), charts y_0_ and y_305_ differ statistically significantly (*p* = 0.05), and charts y_90–305_ and y_305_ differ statistically significantly (*p* = 0.001).

## 4. Discussion

Comparing the curves of milk yield and its components during lactation for different feeding systems is a good way to check whether the diet used has met expectations, with the cow as a biological test, in field conditions. Having access to feed components and very precise computer software for balancing feed rations, many farmers use well-balanced diets (Bach, 2020) [24]. However, it is not always possible to achieve the same results in different herds. The daily amount of concentrated feed and the organization of its use are also relevant.

Comparison of herds with well-balanced diets and very high milk yields (about 11,000 kg of milk for each herd) indicated that the T1 system resulted in by far the highest milk yield. The cows fed the same TMR diet throughout lactation produced more milk on average (42.2 vs. 37.7 kg), and their curves had a higher peak compared with animals fed PMR diets. Moreover, significant differences for peak milk yield were found between feeding management systems (P1 vs. T1) with no division into feeding groups according to days in milk). On the other hand, with the increase in milk production and higher peaks, the contents and curves for protein, fat, and milk urea decreased. In the case of herd T1, the curve for milk urea was similar to the curve for milk yield, with a maximum at about 100 days of lactation for both curves. The similar genetic potential of the herds might suggest that the reasons for these differences can be found in the feeding management systems.

A similar conclusion was drawn by Sabbioni et al. (2012), who found a significant difference in milk yields between traditional and TMR feeding [26,27]. Cichocki et al. (2007) found that the use of TMR and PMR systems did not result in a statistical difference in the level of milk production [28]. Several studies have compared TMR with PMR and traditional feeding systems, in which the forage and concentrate components of the diet are offered to cows separately. Bargo et al. (2002) compared three feeding systems (pasture with concentrate, TMR, and PMR) and found that the TMR feeding system resulted in the highest total milk production: cows produced 6.1 kg/day more milk than the cows from the PMR treatment [29]. This finding was similar to the results of the present study, in which dairy cows fed the same TMR ration throughout lactation, with no division into feeding groups, produced 5 kg/day more milk than in the PMR treatment. Gordon et al. (1995) found that feeding a complete diet resulted in 3.04 kg/day more milk than feeding concentrate and silage separately, without altering the milk concentrations of fat and protein [30]. In the present study, an increase in milk production and the highest peak of the lactation curve were observed in the herd receiving the same TMR ration throughout lactation. In the PMR treatment, the contents of protein, fat, and milk urea in the milk were reduced. Lactation curves for milk and milk components in dairy cattle show variation in peak yield and persistency of yield, partially explained by dietary composition and feeding management (Caccamo et al. 2012) [31]. Cabrita et al. (2007) [32] observed that low-protein, low-starch diets decreased dry matter intake and milk production in mid-lactation cows, but milk production responded to increases in dietary CP, starch, or both. Based on the meta-analysis performed by Hristov et al. (2002) [33], starch (energy content) and forage quality significantly affected herd curve traits, whereas Oba and Allen (2003) [34] observed that cows in early lactation fed a high-starch diet (32%, DM) versus low-starch diets (21%, DM) produce more milk and protein. Ikonen et al. (2004) found that milk yield was negatively correlated with fat (r = –0.25) and protein (r = –0.27) percentages in milk [35]. Moreover, Bargo et al. (2002) concluded that milk yield and milk chemical composition curves were most likely to respond to TMR in studies involving high-yielding cows (>28 kg/day) in early lactation [29].

MU (milk urea: MU = MUN × 21.4) is used in Europe and milk urea nitrogen (MUN) in North America as a tool for monitoring diets (Siachos et al. 2017) [36]. Variance in MU has been shown to be related to the ratio of dietary CP to energy, extended CP degradation in the rumen and the amount of ammonia in excess of microbial N requirements, and protein or energy intake in relation to feeding standards (Nousiainen et al. 2004) [37]. After a meal, microbial degradation of dietary protein is likely to cause an increase in the concentration of rumen ammonia which, due to the transport of rumen ammonia to the blood and the subsequent conversion of blood ammonia into urea by the liver, is followed by rising in plasma urea nitrogen, and due to diffusion between blood and milk as MU [38]. However, diets with a high rapidly degradable carbohydrate fraction may result in a decrease of rumen ammonia immediately after feeding, because the degraded protein in the rumen to ammonia is utilized for microbial protein synthesis [38]. In the current study, in the case of herd T1, the curve for milk urea was similar to the curve for milk yield, with a maximum at about 100 days of lactation for both curves, which could probably mean that this feeding strategy, where cows fed the same TMR diet throughout lactation might have the best balance of CP to carbohydrate ratio in the diet and utilization of nitrogen. The above results are similar to those presented by Wood et al. (2003) [39], who reported an increase in MU concentration after peaking of lactation. However, Mucha et al. (2011) [40] showed that the lactation curves of MU did not seem to be affected by the milk yield curve and differences may have been due to management or nutrition strategies. Surprisingly in the current study of other feeding management systems such as P1, T2, and T3, the increase in milk production was not correlated with MU concentration. The results might suggest that after peaking of lactation, CP intake was too high or supply rapidly degradable carbohydrates was too less for the effective microbial protein synthesis, which had an effect on MU contents and curves.

Professional feeding of dairy cows is a very important target based on economic and animal health reasons. Feed intake is characterized as dry matter intake (DMI) to compare diets of variable moisture concentrations. DMI is affected by both animal and feed factors. The weight of cows, milk production, and the stage of lactation or gestation are the major animal factors. In practice, the grouping of cows in nutritional groups is based on milk production, reproductive cycle and the days in milking. The cows are usually grouped monthly, after milk recording. Metabolic processes increase if milk productivity increases in dairy cows. Metabolization of body energy reserves during the early lactation enables the cow to close the gap between the alimentary energy intake and its loss through milk production [41]. Since the alterations in energy reserves have a considerable influence upon the productivity, health, and reproduction of dairy cows [42], the monetarization of optimal management of energy reserves is obviously needed. Indicators, which characterize dairy cows metabolic processes is body condition score (BCS). The use of a body condition scoring system is more useful than the measurement of body weight (BW) in feeding. BCS has been widely recommended as a method of evaluating the nutritional management of dairy cows [43]. It is a management tool used to elucidate if rations meet animal needs or not. Feeding a cow according to its needs leads to optimal performance. Stefańska et al. (2016) defined BCS as an indicator of how well the animal maintains energy reserves, reflective of the relationship between nutrition and milk production in a herd. Additionally, the production of cows correlates with their body condition which is a wide and effective method to evaluate the nutritional management of dairy cows [44]. Optimal BCS of dairy cows is essential to obtain elite herd and quantity milk production because thin or fat cows may have a greater risk of lower milk yield and higher milk somatic cell counts [45]. Additionally, Atasever et al. (2017) found that cows with lower than 3.0 BCS at calving showed lower milk production [46]. The highest lactation curve, greatest peak and least persistency were obtained from cows calving at BCS 3.25. On the other hand, over-conditioned animals, especially at the end of lactation or under-conditioned animals especially at the beginning of lactation, would have a health problem. Agenas et al. (2003) reported that, at the peak of lactation, the energy needs exceed the energy supply, which generated negative energy balance (NEB) and in consequence decrease BCS and metabolic disease occurrence [47]. On the other hand, after the peak of lactation, the energy needs decreasing, which also is associated with decreasing the milk yield and lactation curve. In this case, if the diets are not well balancing (too high-energy diet level in compiling to the milk production) it might affect improving the BCS in late-lactation dairy cows and then during the dry-off period, which has a negative effect because excessive energy intake prepartum leads to metabolic problems and decreases DMI in the following lactation and loss in milk production [48].

The milk yield curves were influenced by the herd management systems, including the culling rate. The management method in the T1 herd increased average milk production by about 1586.87 kg per primiparous cow. According to Zając-Mazur (2007), an increase in the culling level contributes to progress in the herd through a faster change of generations [49]. Culling for economic reasons increases the utility and breeding value of the whole herd. A study by Rogers et al. (1988) on the American population demonstrated that the optimal percentage of herd culling for farms should be within the range of 25–35% to achieve an economic profit [50]. In Europe, the culling rate is lower; Bergk and Swalve (2011) report that more than 10% of primiparous cows are eliminated from the herd in Germany during the first 300 days and more than 20% within 450 days after calving [51]. However, many other studies demonstrate that in order to achieve satisfactory production results, a cow should be used as long as its productivity is sufficient to cover the expenses incurred for its rearing and maintenance. Juszczak et al. (2003) estimated that the cost of milk produced by cows used only for the first three lactations is equal to its market value, and only when used for a longer period do they begin to generate profit [13]. The authors also state that the main source of economic losses lies in the cost of milk production from primiparous cows used only up to 200 days, especially those culled during the first 100 days of lactation. This effect is exactly the opposite of that observed in our study, which showed that earlier removal of a low-yield animal from the herd and its replacement with an animal meeting the productivity criterion increases the production of the entire herd, with better economic results. This has also been confirmed by Borkowska and Januś (2009), who found that the milk performance of cows was affected by the yield in the first lactation [14]. By far the highest values, especially in the case of yield per day of milk use, were found for the most productive primiparous cows. At the same time, the lower yield of the primiparous cows was found to be associated with a higher percentage of milk fat and a lower percentage of protein.

## 5. Conclusions

To sum up, the best production results in combination with a favourable lactation curve were obtained in herd T1, in which cows were fed in the TMR system with no division into feeding groups according to days in milk. A high rate of culling of cows that does not meet the high production criterion in the first stage of lactation increases the annual milk yield of the herd.

## Figures and Tables

**Table 1:** General information on the herd and the composition and nutritional value of the diets.

	Herds ^1^							
	T1	P1	T2			T3		
General information								
Number of cows in herd	1262	369	749			335		
Number of primiparous cows/farm	779	142	459			282		
Milk production (kg)/farm/per cow	12,778	10,934	11,505			11,346		
Milk fat (%)/farm/per cow	3.41	3.79	3.83			3.65		
Milk protein (%)/farm/per cow	3.25	3.36	3.38			3.23		
Calving interval (days)	409	397	418			417		
Milking system	Rotary	Automatic (robot)	Fish bone			Fish bone		
Diet during lactation	TMR	PMR ^2^	FRESH ^3^	TMR I ^4^	TMR II ^5^	FRESH	TMR I	TMR II
Average dry matter intake (kg, DM)	23.4	22.2	21.4	23.6	22.5	20.4	22.6	21.5
Ingredient, (% DM)								
Maize silage	30.9	32.7	28.5	33	39.2	26.5	28.3	42.3
Alfalfa silage		23.8	15.9	8.4	8.8	16	13.4	16.7
Sugar beet pulp, ensiled		8.0		4.2	5.3	8.1	6.9	9.0
Grass silage	11.8		9.5			8.2		6.3
Brewer’s grain silage								
Hay				2.4	2.2			
Straw	3.9	9.6	9.3	1.7	1.8	8.2	6.2	
Maize grain, ensiled		5.5	4.5	8.1	7.2	5.8	9.7	
Maize grain			4.7	6.8	3.5	5.9		
Maize husks							6	
Barley grain		4.5	5.3	5.1	5.3	4.1	9.7	13.1
Wheat grain		4.5		5	5.2			
Triticale grain								
Rapeseed meal		5.5	4.2	9.2	8.5	3.3	6.4	5
Soybean meal		1.9	7.9	9.1	8.1	6.1	6.1	4.9
Sugar beet pulp, dry	10.7			1.7				
Mineral and vitamin mix ^6^		4.0	5.7	2.3	2.4	4.4	3.3	2.7
Molasses	3.9		4.5		2.1	3.4	2.6	
Inert fat				1.9			1.1	
Calcium carbonate				0.6			0.3	
Rock salt				0.3	0.2			
Lactasan ^7^	38.8							
Urea				0.2	0.2			42.3
Nutrient composition (%)								
DM—dry matter	44.8	40.4	49.3	49.2	45.3	49.3	42.0	42.4
ME (Mcal/kg) ^8^	2,91	2.61	2.60	2.90	2.60	2.60	2.85	2.60
CP—crude protein	16.5	15.5	15.2	17.1	17.1	15.2	15.9	16.6
NDF—neutral detergent fibre	29.5	39.2	31.3	28.1	30.1	31.3	28.8	33.2
ADF—acid detergent fibre	17.3	24.2	20.2	16.3	17.8	20.2	17.8	19.2
NFC—non-fibre carbohydrates	44.9	33.9	41.3	42.9	43.3	41.3	44.1	40.7
Starch	24.7	17.3	20.7	24.7	24.8	20.7	25	24.3
Ether extract	3.3	2.8	2.2	2.9	2.4	2.2	3.3	2.4
Ca	0.97	0.97	0.97	1.04	0.87	0.97	0.74	0.75
P	0.42	0.39	0.42	0.42	0.41	0.42	0.43	0.42
K	1.37	1.71	1.83	1.54	1.60	1.83	1.60	1.70
DCAD meq/100g SMDietary cation–anion difference	28.21	25.97	30.50	20.60	22.42	30.50	28.21	28.28

^1^ Herds—T1—one TMR throughout lactation; P1—one PMR throughout lactation; T2 and T3—three feeding periods: FRESH, TMR I and TMR II, according to days in milk; ^2^ PMR—partial mixed ration; ground concentrates were supplied in the feed bunk of the milking robots: FRESH concentrate (0–40 day) containing 21.8% CP, 26.7% starch and 12.8% NDF; lactation period concentrate (41–305 day) consisting of a mixture of two concentrates in a 3:1 ratio, containing 19.5% CP, 31.6% starch and 13.5% NDF and 23.1% CP, 22.9% starch and 11.3% NDF, respectively; ^3^ FRESH—feeding period from 7 day before calving to 30 day of lactation; ^4^ TMR I—total mixed ration I; feeding period from 31 day to 100 day of lactation; ^5^ TMR II—total mixed ration II; feeding period from 101 day to 305 day of lactation; ^6^ Mineral and vitamin mix: composition—21.5% Ca, 4.0% P, 6.5% Na, 5.5% Mg, 1200 mg/kg Cu, 4000 mg/kg Mn, 15 mg/kg Co, 10,000 mg/kg Zn, 60 mg/kg Se, 1,200,000 jm/kg vitamin A, 180,000 jm/kg vitamin D, 6000 jm/kg vitamin E; ^7^ Lactasan—supplementary feed containing 27% CP, 23% starch and 6.0 % crude fibre and vitamins and macro- and microelements. ^8^ Calculated according to NRC (2001).

**Table 2:** Herd T1 Wood function parameters and actual milk yields per x days of milking with predicted yield for 305 days.

	Parameters			Actual Yield	Predicted Yield
x–d	a	b	c	Area under curve (0–x day)	Area under curve (0–305 day)
30	15.0170	0.2425	−0.0013	833.222	18,036.89
60	12.5080	0.3321	0.0026	1999.796	12,258.44
90	11.0229	0.3903	0.0047	3243.969	10,430.16
120	11.9886	0.3561	0.0038	4513.761	10,997.52
150	12.1962	0.3491	0.0036	5771.006	11,146.57
180	12.9338	0.3273	0.0032	6986.659	11,309.45
210	13.3877	0.3152	0.003	8172.895	11,386.48
240	13.1579	0.321	0.0031	9271.689	11,333.51
270	13.4694	0.3132	0.003	10,337.96	11,347.22
305	13.7833	0.3056	0.0029	11,367.83	11,367.83

**Table 3:** Milk yields (kg) depending on the number of milking days—actual data (0–x_i_) and predicted data (0–x_305_).

Milking Period (0–x_i_^1^)	Number of Milkings	Number of Cows	Yield in Period 0–x_i_	Yield in Period 0–x_305_
0–305	910	91	11,462.84	11,462.84
0–270	513	57	10,236.95	11,156.88
0–240	352	44	9602.265	11,478.32
0–210	434	62	8303.107	11,116.52
0–180	390	65	6779.897	10,653.34
0–150	255	51	5639.378	10,953.72
0–120	164	41	4622.313	9985.867
0–90	195	65	3356.83	9717.556
0–60	124	62	2138.613	5610.461
0–30	90	90	759.2602	7109.765

^1^ x_i_–final day of milking.

## Data Availability

Data is contained within the article.
